# Impact of climatic and water quality parameters on Tilapia (*Oreochromis niloticus*) broodfish growth: Integrating ARIMA and ARIMAX for precise modeling and forecasting

**DOI:** 10.1371/journal.pone.0313846

**Published:** 2025-03-13

**Authors:** Mohammad Abu Baker Siddique, Balaram Mahalder, Mohammad Mahfujul Haque, A. K. Shakur Ahammad

**Affiliations:** 1 Department of Fisheries Biology and Genetics, Faculty of Fisheries, Bangladesh Agricultural University, Mymensingh, Bangladesh; 2 Department of Aquaculture, Faculty of Fisheries, Bangladesh Agricultural University, Mymensingh, Bangladesh; ICAR-Indian Agricultural Statistics Research Institute, INDIA

## Abstract

This study aims to assess the impact of climatic factors and water quality parameters on the growth of tilapia broodfish and develop time series growth models using ARIMA and ARIMAX. Three years longitudinal data on tilapia growth, including length and weight were collected monthly from February 2021 to January 2024. Climatic data were obtained from the Bangladesh Meteorological Department, while water quality parameters in the broodfish pond were measured daily on-site. Key variables such as air temperature, humidity, rainfall, solar intensity, water temperature, dissolved oxygen (DO), pH, and ammonia, showed fluctuation in the ponds. The highest growth rate (5.93%) occurred in April, and the lowest (0.023%) in December. Overall, tilapia growth in weight followed an exponential trend, while the percent growth rate exhibited a seasonal pattern. Pearson correlation analysis indicated a significant association between growth increments and water quality parameters. The ARIMA (3,0,3) model predicted a consistent upward trend in tilapia weight from February 2024 to January 2027. If the pattern continues, the estimated weight of tilapia will reach 803.58 g by the end of January 2027, a 17.05% increase from January 2024, indicating a positive outlook for broodfish health and production. However, the ARIMAX (1,1,1) model for percent weight gain revealed seasonal fluctuations that were strongly influenced by water temperature and solar intensity. Over the three-year period, forecasts indicated a downward trend in percent weight gain during the first year, followed by an upward trend in the second and third years. This indicates the influence of seasonal changes on percent weight gain. The simulation behaviors were consistent with the forecasted trend. These findings have important implications for planning and managing tilapia broodfish production, highlighting the need to consider environmental factors in future aquaculture management.

## 1. Introduction

Tilapia farming in Bangladesh offers numerous advantages, notably in addressing malnutrition and bolstering food security due to its provision of nutritious and affordable protein [1,2]. It simultaneously provides income generation and uplifts livelihoods for rural communities, creating job opportunities and contributing to poverty alleviation [3,4]. Furthermore, tilapia farming boasts environmental benefits, including adaptable farming systems and efficient resource usage [5,6]. The increasing demand for tilapia in both domestic and international markets present opportunities for economic growth and foreign exchange earnings. Its resilience to climate change makes it a suitable choice for regions susceptible to environmental challenges, diversifying aquaculture practices and positively impacting rural communities and the aquaculture industry as a whole [3,7].

The production of high-quality tilapia broodfish is essential to meet the needs of tilapia farmers and enhance the sector in Bangladesh [8]. Fish hatcheries play a crucial role in this process by focusing on traits like growth rate, disease resistance, and market suitability through selective breeding [9]. The availability of quality broodfish, coupled with their emphasis on disease management through stringent biosecurity measures and health monitoring, stimulates the growth of the tilapia farming sector and leads to increased production [10–13]. In hatcheries, maintaining the growth, development, and health of broodfish is crucial for successful seed production [14]. Broodfish selection is based on desirable genetic traits, and their health is critical for passing these traits to their offspring [11]. Healthy broodfish, selected for desirable genetic traits, play a crucial role in passing those traits to their offspring, ensuring improved yields and traits in future generations through disease prevention and regular health assessments, leading to healthier fry with optimal mating behaviors and higher egg quality [15–17]. Proper nutrition, disease control, and environmental conditions for broodfish are essential to increase their reproductive performance and seed production [18,19]. The health of broodfish has a direct impact on the quality of their offspring, with unhealthy broodfish potentially producing weak or deformed fry, thus diminishing productivity, while healthy broodfish contribute to sustainable production of high-quality seed stock by producing offspring with superior survival rates, growth, and overall fitness [15,20,21]. While private hatcheries have seen growth in Bangladesh and seed production has gone up, concerns still exist about the quality of seeds, which directly affects the yield of pond-based aquaculture [22–25]. To address these problems, it is vital to prioritize the well-being and health of broodfish in fish hatcheries and aquaculture farms by implementing suitable health management practices. These factors carry significant weight for potential hatchery operators, aquaculture experts, and relevant parties, not just within Bangladesh but also at an international level.

The increase in fish growth, both in terms of length and weight, is indeed influenced by various factors within the pond environment [26]. Water quality parameters such as temperature, oxygen levels, pH, and water clarity play a critical role in fish growth [27]. Fish are ectothermic, meaning their body temperature is regulated by the surrounding water [28]. Optimal water conditions can enhance their metabolic processes and promote overall growth [22,29]. Poor water quality can stress the fish, suppress their appetite, and hinder their growth [30,31]. Climatic factors, including air temperature, humidity, rainfall, and solar intensity, significantly impact water quality parameters in broodfish ponds [22,32,33]. Elevated air temperatures can lead to increased water temperature, reducing dissolved oxygen levels [34–36]. Temperature fluctuations may stress aquatic organisms, influencing pond pH and potentially causing increased acidity [37]. Conversely, lower temperatures may result in alkaline conditions [38]. High humidity reduces evaporation, raising water temperature, while low humidity increases evaporation, potentially lowering water temperature. Humidity impacts dissolved oxygen by affecting photosynthesis rates [39–41]. pH levels are influenced by humidity, promoting algae growth in high humidity, consuming ammonia, while low humidity may lead to higher ammonia concentrations. Rainfall cools water and increases dissolved oxygen but may introduce pollutants, elevating ammonia and nutrient levels [42,43]. Rainfall also impacts pH, with acid rain lowering pH and threatening pH-sensitive aquatic organisms [44–46]. Solar intensity influences water temperature, affecting biological processes [47–49]. This can increase dissolved oxygen through enhanced photosynthesis but may also raise respiration and decomposition rates, potentially lowering dissolved oxygen [34]. Solar intensity indirectly influences pH and ammonia by affecting biological processes [22]. High solar intensity can reduce CO_2_ levels, elevating pH, while also releasing ammonia, potentially increasing ammonia levels [50]. The availability and quality of food sources within the pond are key determinants of fish growth [51]. Inadequate or low-quality food can result in stunted growth in fish by failing to meet their energy and nutrient needs for proper development [52]. Genetic factors, such as selectively bred strains with enhanced growth traits, can play a pivotal role in determining the growth rate of individual fish within populations [53]. The number of fish present in the pond can affect growth rates. Overcrowding can lead to competition for food and resources, potentially slowing down fish growth [54,55]. So, maintaining an appropriate stocking density is crucial for optimal growth [55]. While these factors can lead to variations in the rate of length and weight increments among individual fish or across different time periods, the total length and total weight of the fish population typically increase over time due to a cumulative effect [56]. As long as the pond environment is well-managed, with suitable water quality and food availability, and genetic factors are favorable, the fish population as a whole tends to grow larger as new individuals are added through reproduction, and existing fish continue to grow [57,58]. However, the fluctuation in the seasonal growth rate of fish is a result of a multifaceted interplay of various factors such as temperature, oxygen levels, pH, water clarity, food availability, reproductive cycles, and the overall dynamics of water quality. The literature stated above meaning that there is a complex relationship existed between climatic factors, water quality parameters and fish growth in the pond system. Grasping the details of these connections is essential for fish farmers and aquaculture professionals to fine-tune management strategies, tackle the challenges, and foster enduring and sustainable growth of fish at hatcheries and aquaculture farms level. In light of the current national and global climate context, it is essential to understand and address these emerging phenomena. This knowledge can help to tackle present challenges and inform future aquaculture practices.

The utility of ARIMA (Auto Regressive Integrated Moving Average) and ARIMAX (Auto Regressive Integrated Moving Average with Exogenous Inputs) models in aquaculture lies in their capacity to accurately predict complex time-dependent patterns in fish growth. ARIMA models are renowned for capturing the dynamics of growth by analyzing historical data to identify trends and seasonal patterns, thus enabling precise forecasts. These models have been instrumental in managing and understanding growth dynamics by predicting growth rates and identifying fish with desirable growth traits, contributing to improved breeding programs and enhancing the genetic potential of future offspring [59–62]. The precision of these models facilitates well-informed stocking decisions, optimizing resource allocation, reducing wastage, and improving resource utilization efficiency. ARIMAX extends this capability by incorporating external variables, such as climatic and water quality parameters, to refine predictions. This makes ARIMAX particularly useful for aquaculture, where external environmental factors significantly influence growth rates [63]. This forecasting not only aids in market planning by anticipating offspring availability and enabling effective marketing strategies but also consistently meets customer demands. In the broader context, ARIMA and ARIMAX models’ versatility makes them applicable in diverse contexts, demonstrated by their use in forecasting fish production in Tamil Nadu, Assam, Odisha, and Chilika lagoon in India, and in agriculture for crops like cotton, rice, and wheat [63,64]. These models offer valuable insights and forecasts, guiding decision-making and planning within the fisheries and agriculture sectors.

Therefore, this study focuses on the multifactorial relationship between climatic and water quality parameters affecting the growth of tilapia broodfish. It aims to predict their growth using ARIMA and ARIMAX models, considering external factors. There is a lack of comprehensive literature on the correlation between these parameters and tilapia broodfish growth, especially with a longitudinal perspective. Moreover, very few articles integrate ARIMA and ARIMAX models for this purpose. By examining these multifactorial impacts over time, this research seeks to fill this gap and forecast tilapia broodfish growth. The findings will provide valuable insights for strategic planning and decision-making in tilapia production, both in Bangladesh and worldwide.

## 2. Materials and methods

### 2.1. Ethical statement

This study encompassed the gathering of fish from both ponds and hatcheries, in addition to performing various associated activities. All protocols that encompassed animal handling adhered rigorously to established scientific protocols and received prior approval from the Animal Welfare and Ethics Committee at Bangladesh Agricultural University (BAU), with the reference code BAURES/ESRC/FISH-11/2022.

### 2.2. Study area

This study took place in a tilapia broodfish pond located in Dhala, Trishal, Mymensingh, Bangladesh. The study site is situated approximately 37 kilometers to the south of Bangladesh Agricultural University in Mymensingh, Bangladesh. The study was conducted over the period from February 2021 to January 2024.

### 2.3. Collection and domestication of broodfish

Three earthen ponds, each covering an area of 35 decimals and with a depth of 4 feet, underwent drainage and maintenance procedures. Lime was applied to the pond bottoms and embankments to mitigate potential harmful pathogens. Following a week’s interval, the ponds were refilled with water. Once the water exhibited a greenish color after two to three days, 7000 broodfish of *O. niloticus* were introduced into each pond in January 2021. These broodfish, procured from a reputable source, displayed an average length, weight, and age of 28.97 cm, 509.33 g, and 2 years, respectively. A male-to-female sex ratio of 40:60 was maintained within each pond. Utilizing commercial floating feed with 38% protein content, the broodfish were fed twice daily at a quantity averaging 3% to 5% of their body weight. Throughout the study period, systematic random sampling was employed to monitor broodstock health, physical attributes, growth trends, disease prevalence, and pond productivity.

### 2.4. Assessment of climatic and water quality parameters

Daily climatic data, including air temperature, humidity, rainfall, and solar intensity, were gathered from the Bangladesh Meteorological Department and averaged on a monthly basis. This data was then used for longitudinal analysis in conjunction with water quality parameters and tilapia growth. Essential water quality parameters, including water temperature, dissolved oxygen (DO), pH, ammonia, and water transparency, were recorded daily using specialized instruments: a SMART Sensor temperature meter (SMART Sensor AR 867), a DO meter (Lutron DO-5509), a compact pH meter (pH-107), ammonia measured with an ammonia test kit, and water transparency determined using a sechi-disk throughout the study. Subsequently, the climatic and water quality parameters were averaged to facilitate their presentation and interpretation as crucial elements of the study.

### 2.5. Assessment of growth of tilapia broodfish in traditional pond

From each of the three replicates, a total of ten fish, comprising an equal distribution of males and females, were chosen. The length and weight of the fish were measured using a traditional wooden scale and digital weight balances for weight measurement. The average length and weight of the fish were computed for each replicate during every sampling occasion. To determine the parameters such as length gain (%) (equation 1), weight gain (%) (equation 2), feed conversion ratio (FCR) (equation 3), and specific growth rate (SGR) (equation 4), the following, adapted from a prior study [65] were used.


$$\%lengthgain=\;\left(Meanfinallength-Meaninitiallength\right)/Meaninitialfishlength\times \;100$$ (1)

$$\%weightgain=\;\left(Meanfinalweight-Meaninitialweight\right)/Meaninitialfishweight\times \;100$$ (2)

$$Foodconversionratio\left(FCR\right)=FeedFed/Liveweightgain$$ (3)

$$Specificgrowthrate\left(SGR\right)\left(\%/day\right)=\;InW_{2}-InW_{1}/T_{2}-T_{1}\times 100$$ (4)

where, W_1_ =  Initial live body weight (g) at time T_1_ (day).

W_2_ = Final live body weight (g) at time T_2_ (day).

### 2.6. Correlation between fish growth and water quality parameters

Pearson correlation analysis was carried out to explore the relationship between tilapia broodfish growth parameters (specifically, length and weight increments denoted as “x” and “y,” respectively) and various water quality parameters averaged monthly over a specified three-year period. Data on tilapia growth parameters, climate, and water quality were meticulously collected in a longitudinal manner. Rigorous procedures were implemented for data collection, cleaning, and validation to ensure high-quality data.

The Pearson correlation coefficient (often denoted as “r”) was calculated using the following equation 5:


$$r=\left[n\sum \left(xy\right)-\sum x\sum y\right]/\surd \left[n\sum \left(x^{2}\right)-{\left(\sum x\right)}^{2}\right]\left[n\sum \left(y^{2}\right)-{\left(\sum y\right)}^{2}\right]$$ (5)

here

- “n” represented the number of data points.
- “x” and “y” were the variables being correlated (in this case, tilapia broodfish growth and water quality parameters).
- “Σ” denoted summation, so the sum of products of “x” and “ y,” the sum of all “x” values, the sum of all “y” values, the sum of squared “x” values, and the sum of squared “y” values were calculated.

Once these values were determined, they were used to calculate the Pearson correlation coefficient “r.” The resulting “r” value indicated the strength and direction of the correlation between the two variables. The strength of Pearson correlation coefficients provided insight into the degree of linear association between variables.

A previous study reported on the scaling of Pearson’s correlation [66]. A robust positive or negative relationship became apparent when there was a very high correlation, falling within the range of 0.80 to 1.00. In such cases, changes in one variable were highly indicative of corresponding changes in the other. When the correlation was high, within the range of 0.60 to 0.79, it signified a pronounced linear connection. Moderate correlations, ranging from 0.4 to 0.59, suggested a moderate level of association, while lower correlations in the range of 0.20 to 0.39 hinted at a more subtle link. Very low correlations, ranging from 0 to 0.19, implied a minimal linear relationship. It’s important to remember that correlation didn’t imply causation, and interpretation depended on context. Standard statistical analysis typically used a 95% confidence level (alpha =  0.05), ensuring 95% confidence in the results. Statistical software assisted in handling complex calculations.

### 2.7. Modeling and forecasting the growth of tilapia broodfish with combination of ARIMA and ARIMAX

In this study, we utilized ARIMA and ARIMAX models, incorporating potential exogenous factors. The forecasting process involved the use of the Box-Jenkins methodology, a widely recognized and significant approach for time series forecasting [67]. This approach relies on the analysis of longitudinal time series data, which is used as a powerful tool in various aspects [68–70]. The ARIMA and ARIMAX statistical models were employed to analyze and forecast the longitudinal time series data of tilapia broodfish growth over the course of a year in a specific study area. These models were effective in capturing any linear associations between previous and current data points, as well as accounting for any errors or residuals within the data.

This modeling approach comprises three components: the AR (p) component, which establishes connections between past and present values; the I (d) component, addressing non-stationary aspects; and the MA (q) component, modeling the relationship between current and past errors or residuals. The model fitting process involves four steps: identification, estimation, diagnostic checking, and forecasting. Before proceeding with these steps, ensuring the stationarity of fish length and weight data is crucial. The Augmented Dickey-Fuller (ADF) test assesses data stationarity, yielding a p-value below the predefined significance level of 0.05. In cases of non-stationarity, visual examination identifies trends or seasonality patterns, and data differencing is iteratively applied until the ADF test confirms stationarity. Additionally, a normal probability plot distribution serves as an additional check for stationarity.

Subsequently, an ARIMAX model, extending the ARIMA model by incorporating exogenous variables like water temperature and solar intensity, is employed for predicting future values of the dependent variable based on independent variables and past values. ARIMAX (Auto-Regressive Integrated Moving Average with exogenous variables) is a time series forecasting model that extends ARIMA and predicts future values of the dependent variable by considering both independent variables and past values of the dependent variable. In the context of growth, the model takes into account the percent weight parameter and influential exogenous factors such as water temperature and solar intensity. Before implementing ARIMAX, we first developed an ARIMA model for each parameter using longitudinal time series data on the study area. The overall modeling and forecasting methodology for ARIMA and ARIMAX can be summarized as follows:

#### 2.7.1. Model identification.

The initial stage of ARIMA modeling involved a comprehensive analysis of the autocorrelation function (ACF) and partial autocorrelation function (PACF) based on three years of longitudinal time series data for tilapia broodfish growth. The ACF and PACF plots were crucial for identifying potential models by highlighting significant lags in the data, which correspond to the autoregressive (AR) and moving average (MA) components of the ARIMA model.

The ARIMA model is expressed as ARIMA (p, d, q), where p represents the order of the autoregressive part, d is the degree of first differencing involved, and q is the order of the moving average part.

The autoregressive component AR(p) models the dependency between an observation and a number of lagged observations. It is represented by the equation 6:


$$X_{t}=\mu +\varepsilon_{t}+\theta_{1}\varepsilon_{t-1}+\theta_{2}\varepsilon_{t-2}+\ldots +\theta_{q}\varepsilon_{t-q}$$ (6)

Here, X_t_ is the observed value at time t, μ is the mean of the time series, ε_t_ is the white noise error term at time t, and θ_1_, θ_2_,..., θ_q_ are the parameters to be estimated.

The moving average component (MA(q)) captures the dependency between an observation and a residual error from a moving average model applied to lagged observations. It is expressed in equation 7 as:


$$X_{t}=\;\phi_{1}X_{t\;-\;1}+\;\phi_{2}X_{t\;-\;2}+\;...+\;\phi_{p}X_{t\;-\;p}+\;\varepsilon_{t}$$ (7)

Here, X_t_ is the observed value at time t, ϕ_1_, ϕ_2_,..., ϕ_p_ are the autoregressive parameters, and ε_t_ is the white noise error term at time t. The integration order d represents the number of differencing steps required to make the time series stationary, which is crucial for stabilizing the mean by removing trends and seasonality.

After identifying potential models using ACF and PACF, parameter estimation was performed to determine the optimal values for p, d, and q. The Bayesian Information Criterion (BIC) and Akaike Information Criterion (AIC) were employed to compare models and select the one with the best fit, where lower BIC and AIC values indicate a better balance between goodness of fit and model complexity.

Subsequently, the residuals of the selected model were analyzed to ensure that they were normally distributed with a mean of zero and constant variance. This step confirmed the adequacy of the ARIMA model in capturing the underlying patterns in the time series data, ensuring its reliability in predicting tilapia broodfish growth trends.

#### 2.7.2. Estimation of parameters.

During the second phase of the ARIMA modeling procedure, the parameters for the selected initial models were determined. Typically, the nonlinear least-squares approach, as outlined in a recognized study, was used to estimate the parameters of the ARIMA model [63]. Various metrics, including root mean square error (RMSE), mean absolute percentage error (MAPE), maximum absolute percentage error (MaxAPE), mean absolute error (MAE), maximum absolute error (MaxAE), Bayesian Information Criterion (BIC), and normalized Akaike Information Criterion (AIC) were applied to estimate the parameters and assess the model’s fit with the actual data, as well as to determine the error margin between predicted and observed values. These metrics were considered crucial in evaluating the accuracy of the ARIMA model. The time series data was carefully examined for stationarity, and appropriate differencing was applied as needed during the calibration of the ARIMA model. For model validation, the time series data were split into a training set (80%) and a testing set (20%). The training set was used to build the ARIMA model, while the testing set evaluated the model’s predictive accuracy. The parameters, including AR(p) and MA(q), were identified using autocorrelation and partial autocorrelation functions. The model was then fitted to the training dataset, and diagnostic checks were performed on the residuals to ensure the adequacy of the model. The model was then validated on unseen data, and its predictive accuracy was assessed using performance metrics such as RMSE, MAPE, MaxAPE, MAE, MaxAE, BIC and AIC. Iterative refinement, including adjustments to parameter values, was undertaken until a well-calibrated and validated ARIMA model was achieved, ready for reliable predictions on new observations. The model with the best forecasting ability has the smallest error criterion value [71]. The following equations (8 to 14) express the values of RMSE, MAPE, MaxAPE, MAE, MaxAE, BIC and AIC, respectively.


$$RMSE=\sqrt{\frac{1}{n}\sum_{t=1}^{n}e_{t}^{2}}$$ (8)

where *e*_*t*_ = *A*_*t*_ *- F*_*t*:_ The residual or error at time *t* (difference between the actual value *A*_*t*_ and the forecasted value F_t_). *n*: Number of observations.


$$MAPE=\frac{100\text{\%}}{n}\sum_{t=1}^{n}\frac{\left|e_{t}\right|}{y_{t}}$$ (9)

where *e*_*t*_ =  *A*_*t*_ *- F*_*t*:_ The residual or error at time *t* (difference between the actual value *A*_*t*_ and the forecasted value F_t_). *y*_*t*_: The actual value at time *t*. *n*: The number of observations.


$$\text{MaxAPE}=\text{max}\left(\left|\frac{At-Ft}{At}\right|x100\right)$$ (10)

where: *A*_*t*_ =  Actual value at time *t. F*_*t*_ =  Forecasted or predicted value at time *t*.


$$MAE=\frac{1}{n}\sum_{t=1}^{n}\left|e_{t}\right|$$ (11)

where *e*_*t*_ =  *A*_*t*_ *- F*_*t*:_ The residual or error at time *t* (difference between the actual value *A*_*t*_ and the forecasted value F_t_). *n*: Number of observations.


$$\text{MaxAE}=\max\left(\left|At-Ft\right|\right)$$ (12)

where: *A*_*t*_ =  Actual value at time *t*. *F*_*t*_ =  Forecasted or predicted value at time *t*.

Bayesian Information Criteria (BIC) was introduced in 1978 as


$$BIC=T\prime log\left(\sigma^{2}\right)+\left(p+q+1\right)logT\prime$$ (13)

here, σ^2^ denotes the mean square error and T′ indicates the number of observations used. The model with the lowest BIC value would be the best [63].


AIC=−2LL+2K (14)

where K indicates the number of estimated parameters in the model, L is the maximum value of the likelihood function for the model, and n denotes the sample size.

#### 2.7.3. Diagnostic test of residuals.

The analysis involved examining the autocorrelation function (ACF) and partial autocorrelation function (PACF) of the residuals to verify their adherence to white noise patterns. In cases where the residuals deviated from white noise, additional diagnostic checks were conducted to assess their randomness and normal distribution. The ACF and PACF did not exceed the specified threshold level, indicating a thorough evaluation of the accuracy of the ARIMA modeling.

A verification process was undertaken to ensure the adequacy of the estimated model in representing the series. The normal probability plot and histogram of residuals were examined to assess the normal distribution of the residual dataset. These visual tools provided insights into whether the residuals followed a normal distribution. A close alignment of data points with a straight line in the normal probability plot indicated approximate normality, while the histogram displayed a bell-shaped curve, indicating a distribution consistent with normality. This analysis played a crucial role in determining the adherence of residuals to the assumption of normal distribution, with graphical representations offering a clear depiction of distribution characteristics.

The selection of the most appropriate ARIMA and ARIMAX models was based on specific analysis and context, using criteria such as the lowest values of comparative RMSE, MAPE, MaxAPE, MAE, MaxAE, normalized BIC, and normalized AIC.

#### 2.7.4. Cross correlation.

The analysis involved using the cross-correlation function (CCF) to examine the relationship between water temperature and solar intensity as climatic variables and percent weight gain, considered the dependent variable. The CCF served as a statistical tool to measure the correlation between two pairs of time series: percent weight gain and water temperature, and percent weight gain and solar intensity. The first step was to pre-whiten the variables using previously fitted ARIMA models. This method eliminates or reduces short-term stochastic persistence, which is crucial in identifying the time lag of the independent variable that influences the dependent variable. Pre-whitening has widespread application in examining various geophysical time series variables [72].

Next, the selected climate variables from the second step were incorporated as covariates in the ARIMAX model. The CCF measured the degree of similarity between the two-time series at different lags. The lag represented the time shift between the two-time series, and the CCF values ranged from -1 to 1. A value of -1 indicated a perfect negative correlation, 0 indicated no correlation, and 1 indicated a perfect positive correlation. A positive CCF value suggested that as one time series increased, so did the other, while a negative value indicated that as one time series increased, the other decreased.

#### 2.7.5. Forecasting.

The final phase of ARIMA modeling involves generating forecasts and conducting a comparative assessment of the model’s accuracy against alternative models, using metrics such as RMSE, MAPE, MaxAPE, MAE, MaxAE, Bayesian Information Criterion (BIC), and Akaike Information Criterion (AIC). Forecasts for the sample period are utilized to measure the model’s confidence, while post-sample period forecasts are used for more precise projections in policymaking and other practical applications.

In the ARIMA (p, d, q) model, the future value of a variable is expressed as a linear combination of past values and previous errors, as shown in equation 15:


$$\Delta^{d}+Y_{t}=\emptyset_{0}+\Delta Y_{t}=\emptyset_{0}+\emptyset_{1}\Delta^{d}Y_{t-1}+\emptyset_{2}\Delta Y_{t-2}+\ldots \ldots +\emptyset_{p}\Delta^{d}Y_{t-p}+\varepsilon_{t}+\theta_{1}\varepsilon_{t-1}+\theta_{2}\varepsilon_{t-2}+\ldots ..+\theta_{q}\varepsilon_{t-q}$$ (15)

Here, Y_t_ is the actual value and ε is the random error at t, d refers to the number of differencing transformations required by the time series to get stationary. θ_t_ and θ_j_ are the coefficients, p and q are integers that are often referred to as autoregressive and moving average, respectively. The autoregressive application captured the effect of past observations, while the moving average terms accounted for the impact of past forecast errors. The combination of these components, along with the constant term (Ø_0_), contributed to modeling the time series data in ARIMA framework.

The equation for ARIMAX (Auto Regressive Integrated Moving Average with Exogenous Inputs) is shown below as equation 16:


$$Y_{t}=\emptyset_{0}+\overset{p}{\underset{i=1}{\sum }}\emptyset_{i}Y_{t-i}+\overset{q}{\underset{j=1}{\sum }}\beta_{j}X_{t-j}+\overset{r}{\underset{k=1}{\sum }}\theta_{k}\epsilon_{t-k}+\epsilon_{t}$$ (16)

where,

- *Y*_*t*_ is the dependent variable at time t.
- *ϕ*_0_ is the constant term (intercept).
- *ϕ*_*i*_ are the autoregressive (AR) coefficients, accounting for the past values of *Y*.
- *X*_*t − j*_ are the exogenous variables (external inputs) at time *t − j*.
- *βj* are the coefficients for the exogenous variables *X.*
- *∊*_*t*_ is the error term at time t.
- *θ*_*k*_ are the moving average (MA) coefficients for the lagged errors.
- *p, q,* and *r* represent the number of lags for the AR, exogenous, and MA components, respectively.

The forecasting approach used was one-step-ahead. A rolling one-step-ahead forecast was conducted, where each prediction was made sequentially and updated based on new data. The metrics were calculated using this one-step-ahead testing strategy to ensure the robustness and accuracy of the ARIMAX model in forecasting tilapia percent weight gain.

#### 2.7.6. Simulation.

Simulation has proven to be a valuable forecasting method, particularly in situations where a mathematical model is either inadequate or overly complex for analytical solutions [73,74]. In the context of our ARIMAX modeling and forecasting, simulation was strategically employed to address uncertainty and variability in predictions, thereby enhancing their reliability for decision-making, especially concerning percent weight gain.

This approach became particularly beneficial when multiple sources of uncertainty influenced future outcomes, allowing us to quantify and manage associated risks effectively. To integrate simulation into ARIMAX modeling, we initially obtained forecasted values for percent weight gain, using them to generate future scenarios. This process involved selecting random numbers from appropriate sources to determine the sequence of values for the variable of interest.

Subsequently, a standard mathematical function was fitted to these simulated values. This systematic approach enabled us to explore potential outcomes and assess the likelihood of different scenarios, providing crucial insights for navigating complex and uncertain situations. By employing simulation, we gained a comprehensive understanding of the range of possible outcomes, thus empowering decision-makers with valuable information for strategic planning.

### 2.8. Statistical analysis

Three years of longitudinal time series data, spanning from February 2021 to January 2024, were initially organized, combined, and formatted using Microsoft Excel 2016. Basic descriptive statistics were then conducted on the climate and water quality parameters using Minitab 2019.

The findings were then categorized according to specific criteria. Pearson’s correlation analysis was performed using both Minitab 2019 and SPSS 2023 to model and analyze the time series data with practical implications. To forecast percent weight gain, ARIMA modeling, and simulation were achieved using the NumXL add-ins in version 2016, taking into account influential exogenous factors (ARIMAX). The results were presented based on various indicators and key aspects.

## 3. Results

### 3.1. Determination of climatic and water quality parameters

**Fig 1:**
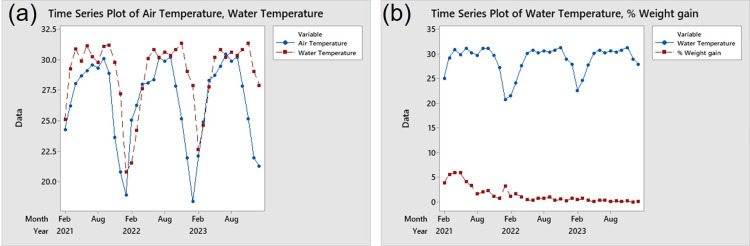
Monthly variation of air temperature, water temperature & % weight gain. Air temperature & water temperature (panel a) and water temperature & % weight gain (panel b).

Over three years course of the study, fluctuations were observed in various climatic factors and water quality parameters which ultimately influenced tilapia growth (Fig 1, panels a & b). In terms of climatic variables, the air temperature ranged from 18.36°C to 30.48°C, humidity levels varied between 46.41% and 85.80%, rainfall showed variability from 0 mm to 485 mm, and solar intensity fluctuated between 2.06 hours and 8.69 hours. Regarding water quality parameters, the water temperature ranged from 19.13°C to 33.20°C, dissolved oxygen (DO) levels varied between 3.30 mg/L and 11.20 mg/L, pH values ranged from 6.74 to 10.32, ammonia concentrations fluctuated from 0 mg/L to 0.333 mg/L, and water transparency varied between 15.37 cm and 27.32 cm.

### 3.2. Assessment of growth of tilapia broodfish in traditional pond system

Table 1 presented the descriptive statistics for tilapia broodfish length and weight throughout the study period, while S1 Fig (panels a to d) offered a graphical representation of the weight data, including histograms, individual value plots, and box plots. The tilapia broodfish began the study with an average length of 27.00 cm and weight of 430.00 g. By the end of the study, their average length had increased to 38.50 cm, and their average weight had risen to 686.54 g. The overall growth pattern for both length and weight followed a steady exponential trend over the study period.

**Table 1:** Descriptive statistics of length and weight of Tilapia broodfish during study period.

Measures	Tilapia Length (cm)	Tilapia weight (g)
Minimum	27.00	430.00
Maximum	38.50	686.50
1st Quartile	31.43	581.20
3rd Quartile	36.49	677.30
Mean	36.00	100.00
Median	34.18	645.00
SE Mean	33.80	620.60
Standard deviation	0.54	11.80
Skewness	-0.50	-1.24
Kurtosis	-0.77	0.74

The inclusion of standard deviation, skewness, and kurtosis in the analysis provided deeper insight into the distribution characteristics of the length and weight data. The standard deviation for length (0.54 cm) indicated a high degree of uniformity and consistency in the growth of the broodfish, whereas the standard deviation for weight (11.80 g) suggested greater diversity and variability. Skewness was used to assess the asymmetry of the data distribution. The slight negative skewness in length data indicated that most fish were slightly longer than the average, with fewer smaller individuals. In contrast, the noticeable negative skewness in weight data suggested that a larger proportion of fish were slightly heavier than the average, with fewer lighter individuals. Kurtosis, which measured the ‘peakedness’ of the distribution, revealed that the length data had a flatter shape, indicating fewer extreme outliers, while the weight data had a more peaked distribution, indicating a higher occurrence of values near the mean with some deviation from normality.

**Fig 2:**
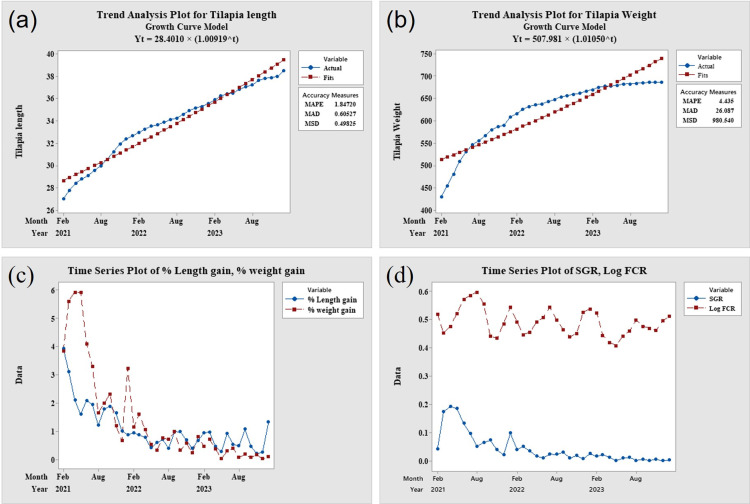
Growth pattern, FCR and SGR of tilapia. (Panel a) exponential trend of fish length, (panel b) exponential trend of fish weight, (panel c) Changing pattern of percent length gain & percent weight gain, (panel d) Changing pattern of SGR & FCR.

Over the three-year study period, monthly variations were observed in percent length gain, percent weight gain, specific growth rate (SGR), and food conversion ratio (FCR), despite a general increase in length and weight (Fig 2, panels a to d). The highest percentage of length gain (3.931%) occurred in February 2021, while the highest percentage of weight gain (5.939%) was recorded in April 2021. Conversely, the lowest percentage of length gain (0.212%) was observed in November 2023, and the lowest percentage of weight gain (0.0233%) was recorded in December 2023. The highest SGR (0.193) was found in April 2021, while the lowest SGR (0.001) occurred in May and December 2023. Meanwhile, the highest FCR (2.96) was observed in August 2021, while the lowest FCR (1.56) was recorded in May 2023.

### 3.3. Correlation between Tilapia broodfish growth and water quality parameters

The analysis of Pearson correlation coefficients revealed several significant relationships among the climatic factors, water quality parameters, and biological variables (Table 2 and S2 Fig). Air temperature showed a moderate positive correlation with rainfall (r =  0.578), suggesting that higher air temperatures were associated with increased rainfall, and a similar correlation with water temperature (r =  0.595), indicating that warmer air led to warmer water. Additionally, there was a moderate positive correlation between air temperature and pH (r =  0.504), implying that higher air temperatures resulted in higher pH levels in the water. Conversely, air temperature was moderate negative correlation with solar intensity (r =  -0.503), meaning that as air temperature rose, solar intensity tended to decrease. Humidity showed a moderate positive correlation with rainfall (r =  0.440), implying that increasing humidity led to more rainfall, and a moderate negative correlation with solar intensity (r =  -0.408), indicating that as humidity increased, solar intensity decreased. Rainfall also exhibited a moderate positive correlation with water temperature (r =  0.478), indicating that increased rainfall was associated with warmer water temperatures, and a higher negative correlation with solar intensity (r =  -0.677), where higher rainfall correlated with lower solar intensity. pH was moderately positively correlated with ammonia levels (r =  0.555), suggesting that higher pH levels coincided with higher ammonia concentrations. It also moderate positive correlation with % weight gain (r =  0.523) and specific growth rate (SGR) (r =  0.501), indicating that higher pH levels were linked to improved growth performance. Ammonia levels showed highly positive correlations with % length gain (r =  0.625) and % weight gain (r =  0.601), suggesting that higher ammonia concentrations were associated with increased fish growth in both length and weight. Additionally, ammonia levels moderately positive correlation with SGR (r =  0.450), reinforcing the association between ammonia and growth rate. Water transparency was moderately positively correlated with % length gain (r =  0.410), implying a relationship with pond productivity. The % length gain and % weight gain were highly correlated (r =  0.765), indicating that fish gaining more length also gained more weight, and both were positively correlated with SGR (r =  0.635 for length gain and r =  0.968 for weight gain), highlighting that greater growth in length and weight corresponded with higher specific growth rates. These findings indicated significant interactions between climatic factors, water quality parameters, and biological responses, highlighting the importance of these variables in understanding pond ecosystem dynamics.

**Table 2:** Pearson correlations between Tilapia broodfish growth and water quality parameters.

	Air Temperature	Humidity	Rainfall	Solar intensity	Water temperature	DO	pH
Humidity	0.197						
Rainfall	**0.578**	**0.440**					
Solar intensity	**-0.503**	**-0.408**	**-0.677**				
Water temperature	**0.595**	0.162	**0.478**	-0.384			
DO	0.126	0.130	0.317	-0.113	-0.009		
pH	**0.504**	0.001	0.215	-0.164	0.167	-0.281	
Ammonia	0.104	0.122	0.158	0.109	-0.029	-0.103	**0.555**
Water transparency	-0.037	-0.020	-0.240	0.162	-0.201	-0.204	0.122
% Length gain	0.028	0.347	-0.170	0.067	-0.063	-0.272	0.378
% weight gain	0.076	0.314	-0.055	0.120	-0.044	-0.133	**0.523**
FCR	-0.058	0.311	0.245	-0.376	-0.061	-0.228	0.338
SGR	0.110	0.303	-0.019	0.090	0.011	-0.125	**0.501**
	**Ammonia**	**Water** **transparency**	**% Length** **gain**	**% weight** **gain**	**FCR**		
Humidity							
Rainfall							
Solar intensity							
Water temperature							
DO							
pH							
Ammonia							
Water transparency	**0.406**						
% Length gain	**0.625**	**0.410**					
% weight gain	**0.601**	0.294	**0.765**				
FCR	0.256	-0.154	0.214	0.276			
SGR	**0.450**	0.187	**0.635**	**0.968**	**0.255**		

[A 95% confidence level (alpha = 0.05) as a standard was considered in statistical analysis. Correlations were considered statistically significant if p ≤ 0.05. Very low correlation: |r| = 0 to 0.19, Low correlation: |r| = 0.20 to 0.39, Moderate correlation: |r| = 0.40 to 0.59, High correlation: |r| = 0.60 to 0.79, Very high correlation: |r| = 0.80 to 1.00].

### 3.4. Modeling and forecasting the growth of Tilapia Broodfish (based on weight) using ARIMA and percent weight gain with ARIMAX

**Fig 3:**
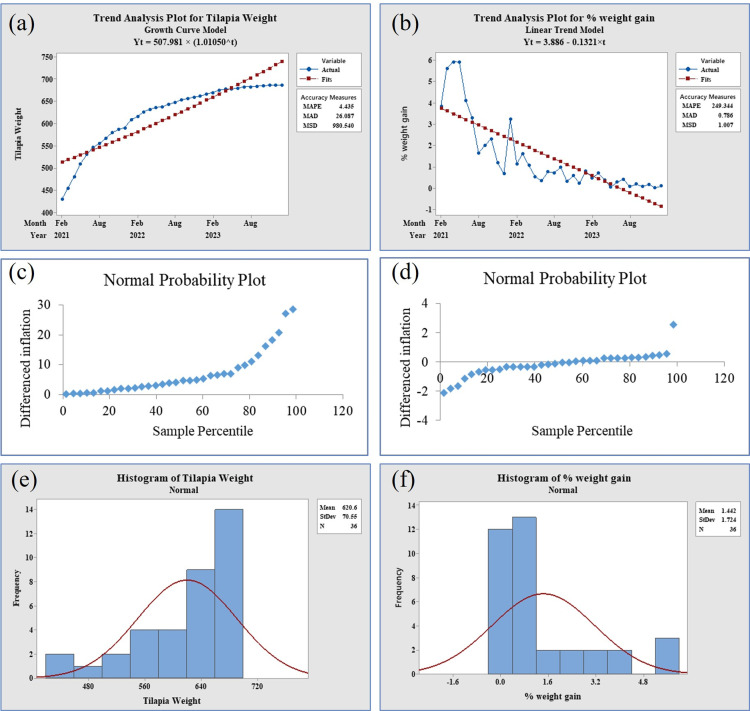
Data stationarity checking of tilapia broodfish growth. (Panel a) trend analysis plot for weight, stationary, (panel b) trend analysis plot for % weight gain, non-stationary, (panel c) normal probability plot of tilapia weight by ADF test, stationary, observations clustered within a low percentile region of the ADF test statistic’s distribution, (panel d) normal probability plot of % weight gain by ADF test, non-stationarity, a substantial number of observations outside the critical region, (panel e) normal histogram of weight, stationary as the data exhibits normal distribution and has a distinctive peak in the center, gradually tapering off, (panel f) normal histogram of % weight gain, non-stationary as it did not exhibit a typical distinct peak in the center and a gradual tapering off.

The Augmented Dickey-Fuller (ADF) tests revealed that the p-value for the weight data of tilapia broodfish was 0.0001, indicating stationarity as it fell below the significance threshold of 0.05. In contrast, the percent weight gain data yielded a p-value of 0.272, signifying non-stationarity as it exceeded the significance level of 0.05. The evidence supporting data stationarity and non-stationarity is further illustrated through trend analysis, normal probability plots, and histograms of tilapia weight (Fig 3, panels a, c & e) and % weight gain (Fig 3, panels b, d & f). Examining the normal probability plots associated with the ADF test, the weight data exhibited observations concentrated in a lower percentile range of the ADF test statistic’s distribution, implying stationarity (Fig 3, panel c). The normal histogram of weight confirms stationarity, as the data exhibits a normal distribution with a distinctive peak in the center, gradually tapering off (Fig 3, panel e). Conversely, the percent weight gain data displayed a significant number of observations beyond the critical region, suggesting potential non-stationarity (Fig 3, panel d). Simultaneously, the normal histogram of % weight gain supports non-stationarity, as it does not exhibit a typical distinct peak in the center and a gradual tapering off, as evident in Fig 3, (panel f).

**Fig 4:**
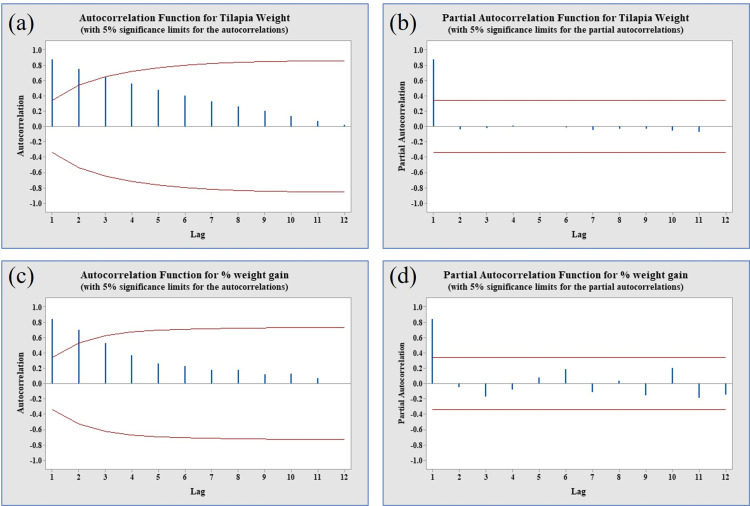
The ACF and PACF of stationarity checking of growth data. (Panels a & b) ACF & PACF of weight with 5% significance limit showed stationary pattern as the plots indicating a rapid decline in correlations, weakening ACF associations with greater lags, and significant PACF spikes primarily at the initial lags. (panels c & d) ACF & PACF of % weight gain with 5% significance limit, showed non-stationary, as the ACF demonstrated a gradual decrease in correlations, with evident connections between the series and its lags, and significant PACF peaks at various lags, suggesting the presence of trends or seasonality.

The analysis of Autocorrelation Function (ACF) and Partial Autocorrelation Function (PACF) plots provided valuable insights into the characteristics of the time series longitudinal data. Specifically, the ACF and PACF plots for tilapia weight data demonstrated stationarity (Fig 4, panels a & b). This stationarity was evident through a rapid decline in correlations, indicating diminishing associations in the ACF with increasing lags, and prominent PACF spikes primarily at the initial lags (Fig 4, panels a & b). These patterns suggest that the weight data is inherently stationary, devoid of persistent trends or seasonality. Consequently, this allows for a straightforward time series longitudinal data analysis without the need for extensive transformations. Conversely, the ACF and PACF plots for percent weight gain data indicated non-stationarity (Fig 4, panels c & d). In this case, the plots often exhibited a gradual decrease in correlations in the ACF, revealing noticeable connections between the series and its lags (Fig 4, panels c & d). Additionally, significant PACF peaks at various lags suggested the presence of trends or seasonality (Fig 4, panels c & d). This non-stationary nature implies that further considerations, such as transformations, may be necessary for a comprehensive analysis of the percent weight gain data.

**Fig 5:**
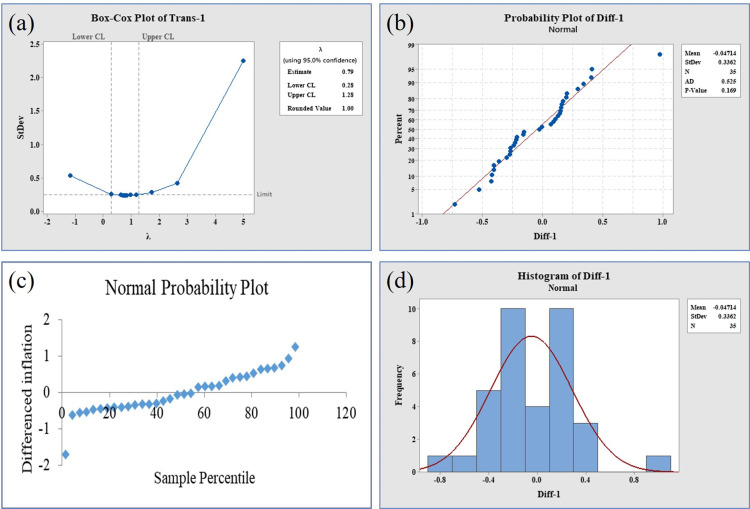
Stationarity of transformed data for tilapia % weight gain. (Panel a) Box-Cox plot where value of λ=1, (panel b) probability plot distribution, support the significant value (p > 0.05), (panel c) normal probability plot by ADF test, observations clustered within a low percentile region of test statistic’s distribution, (panel d) normal histogram, normal bell-shaped distribution and has a distinctive peak in the center, gradually tapering off.

**Fig 6:**
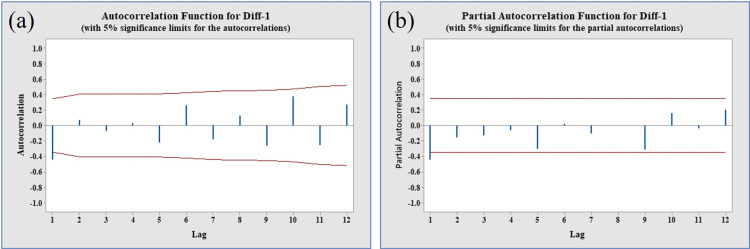
The ACF and PACF of stationary data % weight gain. (Panel a) ACF with 5% significance limit, (panel b) PACF with 5% significance limit.

The investigation revealed dynamic changes in both the mean and variance of the tilapia percent weight gain data over time, suggesting an absence of a consistent pattern in the original data series due to fluctuations in both parameters. To assess the stability of the data compared to the original series, the Box-Cox Plot test was employed for percent weight gain data. The first difference data exhibited a stationary pattern with a λ value of 1 (Fig 5a). This observation was corroborated by various analyses, including trend analysis, the normal probability plot distribution of the Augmented Dickey-Fuller (ADF) test, the normal probability plot based on the Anderson-Darling test (with a p-value of 0.169, exceeding the significance level of 0.05), and the normal histogram with a bell-shaped curve. Collectively, these analyses indicated that the data series achieved stationarity after the first differentiation (Fig 5, panels b, c & d). Similarly, the Autocorrelation Function (ACF) and Partial Autocorrelation Function (PACF) plots also presented visual evidence of a stationary data series (Fig 6, panels a & b).

**Fig 7:**
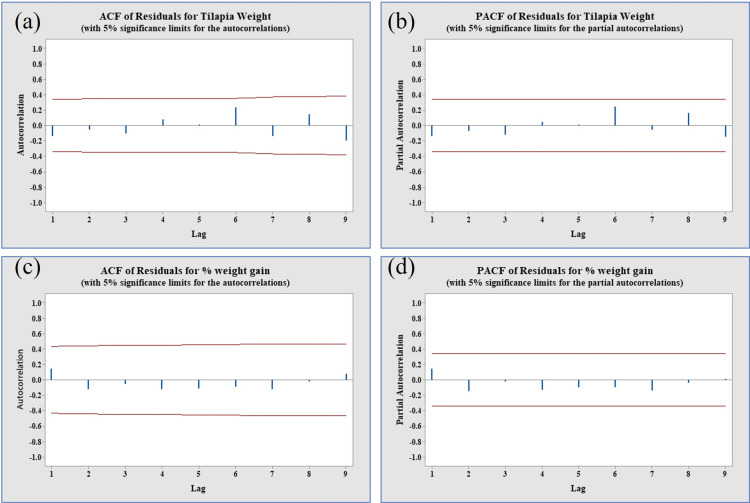
Residual ACF and PACF with normal spike distribution tilapia broodfish growth. Length (panels a & b) and weight (panels **c** & d).

Tables 3 and 4 were also utilized for the evaluation of model performance based on metrics such as RMSE, MAPE, MaxAPE, MAE, MaxAE, normalized BIC and AIC values for the prediction of tilapia broodfish weight and percent weight gain data, respectively. Among the various models considered, ARIMA (3,0,3) exhibited the lowest BIC and AIC values and reasonable lowest values of RMSE, MAPE, MaxAPE, MAE, and MaxAE, making it the preferred model for tilapia broodfish weight. Similarly, for tilapia broodfish percent weight gain data, ARIMA (1,1,1) was identified as the optimal model (as indicated in Table 4). Additionally, an analysis of the Residual ACF and Residual PACF for both cases revealed normally distributed spikes (Fig 7, panels a & b for weight and panels c & d for % weight gain). Furthermore, the forecasted time series displayed no evidence of white noise errors, confirming that ARIMA (3,0,3) for weight and ARIMAX (1,1,1) for percent weight gain were the most appropriate models (Fig 7, panels a & b for weight and panels c & d for % weight gain).

**Table 3:** Suitable model fitting for tilapia broodfish weight data. The model is accepted with minimum BIC and AIC (*) value and considerable other values for the models.

Model	R Square	RMSE	MAPE	MaxAPE	MAE	MaxAE	Normalize BIC	AIC
ARIMA (1, 0, 1)	0.876	26.035	1.821	32.491	9.043	139.698	6.917	232.11
ARIMA (2, 0, 2)	0.906	23.374	1.587	28.278	7.923	121.585	6.900	422.61
ARIMA (3, 0, 3)	0.992	7.205	0.731	5.268	3.957	23.908	4.746	209.05*
ARIMA (4, 0, 4)	0.962	15.971	1.081	17.490	5.492	75.198	6.537	271.75
ARIMA (4, 0, 2)	0.978	11.813	0.928	12.684	4.793	54.536	5.735	429.61
ARIMA (5, 0, 5)	0.993	6.909	0.636	5.266	3.467	23.897	5.060	448.61

**Table 4:** Suitable model fitting for tilapia percent weight gain data. The model is accepted with minimum BIC and AIC (*) value and considerable other values for the models.

Model	R-squared	RMSE	MAPE	MaxAPE	MAE	MaxAE	Normalized BIC	AIC
ARIMA (1,1,1)	0.778	0.837	38.456	159.387	1997.647	2.077	0.051	110.9*
ARIMA (2,1,2)	0.782	0.859	158.597	1946.882	0.567	2.140	0.306	161.37
ARIMA (3,1,3)	0.792	0.870	141.970	1591.480	0.551	2.237	0.534	170.37
ARIMA (4,1,4)	0.800	0.886	158.638	2192.962	0.534	2.128	0.775	180.37
ARIMA (5,1,5)	0.830	0.851	157.532	2144.128	0.489	1.730	0.897	194.37

In Table 5 and S3 Fig (panels a & b), the cross-correlation function (CCF) values of three years of longitudinal data between percent weight gain and changes in water temperature and solar intensity at various time lags are shown. Specifically, for percent weight gain and water temperature, a positive CCF value of 0.106051 was observed at lag -10, suggesting that an increase in water temperature ten-time units ago was associated with a rise in percent weight gain. Positive CCF values at lags -9 (0.129652), -8 (0.061085), -7 (0.066538), -6 (0.071762), -5 (0.043253), -4 (0.047575), 1 (0.072743), 2 (0.130733), 3 (0.118499), 4 (0.121407), and 5 (0.073486) further indicate that increases in water temperature during these periods corresponded to higher percent weight gain. Negative CCF values were identified at lags -12 (-0.031994), -11 (-0.002243), -3 (-0.002345), -2 (-0.065533), -1 (-0.062667), 0 (-0.043898), 6 (-0.026238), 7 (-0.142202), 8 (-0.328966), 9 (-0.474321), 10 (-0.513309), 11 (-0.442831), and 12 (-0.212607), indicating that, after these lags, an increase in water temperature was associated with a decrease in percent weight gain.

**Table 5:** Cross correlation of percent weight gain & water temperature and percent weight gain & solar intensity.

Percent weight gain and water temperature		Percent weight gain and solar intensity	
Lag	CCF	Lag	CCF
-12	-0.031994	-12	0.029156
-11	-0.002243	-11	0.061403
-10	0.106051	-10	0.014667
-9	0.129652	-9	0.010524
-8	0.061085	-8	-0.05207
-7	0.066538	-7	-0.07376
-6	0.071762	-6	-0.05716
-5	0.043253	-5	0.026288
-4	0.047575	-4	0.087745
-3	-0.002345	-3	0.164922
-2	-0.065533	-2	0.239851
-1	-0.062667	-1	0.17355
**0**	**-0.043898**	**0**	**0.120045**
1	0.072743	1	0.090233
2	0.130733	2	-0.08314
3	0.118499	3	-0.20941
4	0.121407	4	-0.25471
5	0.073486	5	-0.223
6	-0.026238	6	-0.09369
7	-0.142202	7	0.060494
8	-0.328966	8	0.159533
9	-0.474321	9	0.202919
10	-0.513309	10	0.234654
11	-0.442831	11	0.167923
12	-0.212607	12	0.109871

Similarly, for percent weight gain and solar intensity, a positive CCF value of 0.029156 was observed at lag -12, suggesting that an increase in solar intensity twelve-time units ago was associated with a rise in percent weight gain. Positive CCF values at lags -11 (0.061403), -10 (0.014667), -9 (0.010524), -5 (0.026288), -4 (0.087745), -3 (0.164922), -2 (0.239851), -1 (0.17355), 0 (0.120045), 1 (0.090233), 7 (0.060494), 8 (0.159533), 9 (0.202919), 10 (0.234654), 11 (0.167923), and 12 (0.109871) further indicate that increases in solar intensity during these periods corresponded to higher percent weight gain. Negative CCF values were identified at lags -8 (-0.05207), -7 (-0.07376), -6 (-0.05716), 2 (-0.08314), 3 (-0.20941), 4 (-0.25471), 5 (-0.223), and 6 (-0.09369), indicating that, after these lags, an increase in solar intensity was associated with a decrease in percent weight gain.

**Fig 8:**
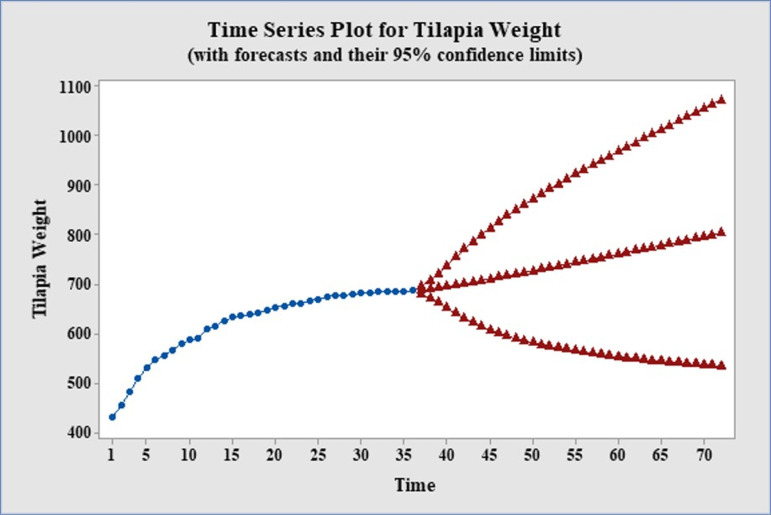
Weight forecasting. Forecasting the tilapia broodfish weight up to the end of January 2027 with ARIMA.

**Fig 9:**
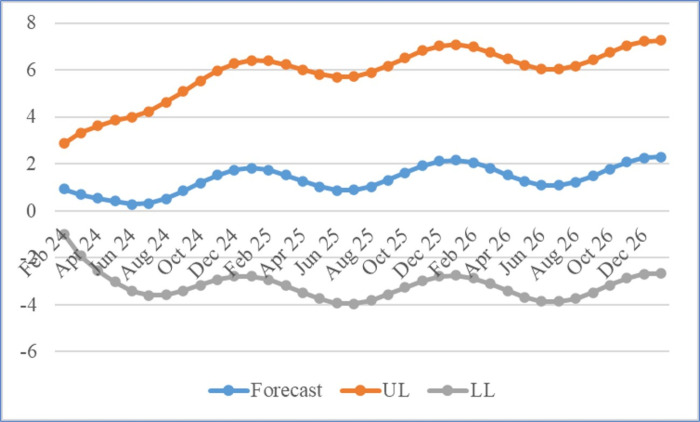
Percent weight gain forecasting. Forecasting the tilapia broodfish percent weight gain up to the end of January 2027 with ARIMAX.

Table 6 and Fig 8 displayed the predicted values for tilapia broodfish weight for the following four years, while Table 7 and Fig 9 showed the predictions for tilapia broodfish percent weight gain. These predictions were found to fall within the 95% confidence limits. The figures illustrated the trends in tilapia weight and % weight gain using the ARIMA (3,0,3) and ARIMAX (1,1,1) models, respectively. The analysis indicated a consistent upward trend in weight, with a projected attainment of 803.58 g by the end of January 2027. On the one hand, the rate of weight gain showed a seasonal up-down pattern, which could be attributed to the influence of water temperature and solar intensity. This demonstrated a notable reduction in percent weight gain in nearly all months compared to the first year’s original data, followed by an upward trend relative to the original data from the second and third years. This pattern also indicated future seasonal fluctuations in the percent weight gain of tilapia broodfish within a traditional pond management system.

**Table 6:** Forecasting of tilapia weight from February 2024 to January 2027 with ARIMA.

		95% confidence level	
Period	Forecast	Lower	Upper
Feb 24	687.968	679.085	696.85
Mar 24	689.803	671.767	707.84
Apr 24	692.074	662.641	721.51
May 24	694.718	651.874	737.56
Jun 24	697.617	641.043	754.19
Jul 24	700.673	631.014	770.33
Aug 24	703.82	622.06	785.58
Sep 24	707.02	614.165	799.88
Oct 24	710.252	607.201	813.3
Nov 24	713.506	601.017	825.99
Dec 24	716.776	595.48	838.07
Jan 25	720.062	590.476	849.65
Feb 25	723.363	585.917	860.81
Mar 25	726.678	581.734	871.62
Apr 25	730.009	577.873	882.14
May 25	733.354	574.292	892.42
Jun 25	736.715	570.956	902.47
Jul 25	740.091	567.838	912.34
Aug 25	743.483	564.915	922.05
Sep 25	746.89	562.169	931.61
Oct 25	750.313	559.583	941.04
Nov 25	753.751	557.143	950.36
Dec 25	757.205	554.839	959.57
Jan 26	760.675	552.658	968.69
Feb 26	764.161	550.594	977.73
Mar 26	767.663	548.636	986.69
Apr 26	771.181	546.78	995.58
May 26	774.715	545.017	1004.41
Jun 26	778.265	543.343	1013.19
Jul 26	781.831	541.753	1021.91
Aug 26	785.414	540.241	1030.59
Sep 26	789.014	538.804	1039.22
Oct 26	792.629	537.439	1047.82
Nov 26	796.262	536.14	1056.38
Dec 26	799.911	534.906	1064.91
Jan 27	803.576	533.734	1073.42

**Table 7:** Forecasting of tilapia percent weight gain from February 2024 to January 2027 with ARIMAX.

		95% confidence level	
Month/Period	Forecast of % weight gain	Upper limit	Lower limit
Feb 24	0.9384	2.8654	-0.9887
Mar 24	0.6969	3.3177	-1.9240
Apr 24	0.5313	3.6215	-2.5589
May 24	0.4137	3.8528	-3.0253
Jun 24	0.2861	3.9958	-3.4237
Jul 24	0.3192	4.2443	-3.6059
Aug 24	0.5258	4.6250	-3.5734
Sep 24	0.8449	5.0864	-3.3966
Oct 24	1.1927	5.5515	-3.1660
Nov 24	1.5230	5.9790	-2.9329
Dec 24	1.7468	6.2837	-2.7900
Jan 25	1.8193	6.4238	-2.7853
Feb 25	1.7366	6.3978	-2.9247
Mar 25	1.5306	6.2395	-3.1783
Apr 25	1.2759	6.0249	-3.4731
May 25	1.0347	5.8175	-3.7482
Jun 25	0.8808	5.6921	-3.9306
Jul 25	0.8857	5.7212	-3.9498
Aug 25	1.0342	5.8900	-3.8217
Sep 25	1.3044	6.1775	-3.5687
Oct 25	1.6276	6.5153	-3.2601
Nov 25	1.9215	6.8216	-2.9786
Dec 25	2.1196	7.0302	-2.7911
Jan 26	2.1639	7.0834	-2.7556
Feb 26	2.0547	6.9818	-2.8724
Mar 26	1.8238	6.7573	-3.1097
Apr 26	1.5276	6.4665	-3.4113
May 26	1.2648	6.2083	-3.6788
Jun 26	1.0972	6.0446	-3.8503
Jul 26	1.0894	6.0402	-3.8614
Aug 26	1.2208	6.1744	-3.7328
Sep 26	1.4774	6.4334	-3.4786
Oct 26	1.7895	6.7475	-3.1686
Nov 26	2.0827	7.0425	-2.8770
Dec 26	2.2621	7.2234	-2.6991
Jan 27	2.2942	7.2567	-2.6683

**Fig 10:**
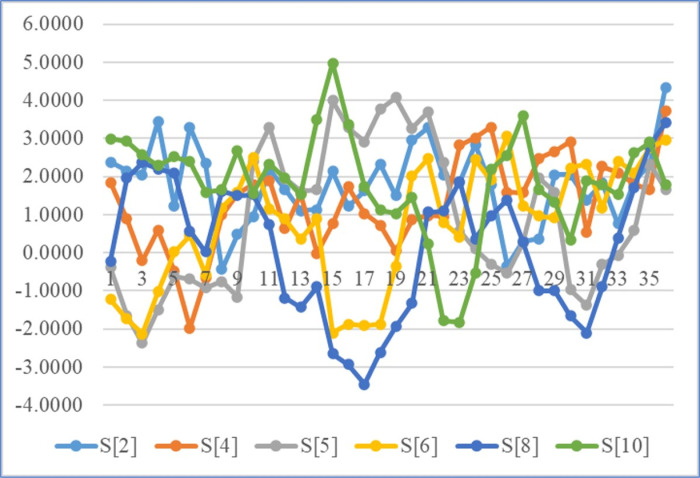
Percent weight gain simulation. Simulation of percent weight gain up to the end of January 2027 with ARIMAX.

In the simulation results, as shown in Table 8 and Fig 10, the percentage weight gain of tilapia broodfish also displayed fluctuations and a clear seasonal trend in accordance with three years original longitudinal data series, strongly supporting the findings obtained from the forecasting analysis. The data indicates a consistent decline in percent weight gain compared to the first year’s data series, followed by an upward trend relative to the original second- and third-year data series. Notably, it is observed that seasonal changes may lead to an increase in percent weight gain during the winter, assuming favorable climatic conditions, optimal water quality parameters, and other factors within the broodfish pond that could positively impact weight gain.

**Table 8:** Simulation of percent weight gain in 10 series up to the end of January 2027 with ARIMAX.

Step	S[1]	S[2]	S[3]	S[4]	S[5]	S[6]	S[7]	S[8]	S[9]	S[10]
Feb 24	2.5493	2.3771	2.9000	1.8393	-0.3777	-1.2270	-3.7716	-0.2206	0.4295	2.9908
Mar 24	2.6174	2.1529	2.2497	0.8920	-1.6505	-1.7363	-3.1443	1.9569	1.5294	2.9290
Apr 24	1.7237	2.0485	2.4115	-0.2107	-2.3643	-2.1345	-1.3495	2.3708	-0.4906	2.5676
May 24	1.2943	3.4556	2.0545	0.5910	-1.4872	-1.0235	-1.3784	2.1986	-0.5035	2.2863
Jun 24	0.9106	1.2182	1.2808	-0.4850	-0.6120	0.0207	-1.4904	2.0883	-0.3607	2.5257
Jul 24	0.9600	3.2980	0.7492	-1.9727	-0.6833	0.4522	-1.3201	0.5754	0.0025	2.3895
Aug 24	2.1136	2.3392	1.5268	-0.6579	-0.9182	-0.5804	-0.7101	0.0200	-0.8778	1.5873
Sep 24	1.2836	-0.4376	1.8092	0.9873	-0.7486	1.1810	-1.4293	1.6157	-1.2867	1.6571
Oct 24	-0.9552	0.4850	2.2769	1.5406	-1.1549	1.5905	0.1242	1.5011	-0.2950	2.6840
Nov 24	-1.4479	0.9548	1.5931	1.7968	2.4054	2.5054	1.4442	1.5207	-0.3020	1.5707
Dec 24	-2.1392	2.1765	2.6711	1.8988	3.2935	1.1534	2.3032	0.7361	0.5346	2.3288
Jan 25	-2.6388	1.6693	2.7411	0.6333	1.9454	0.8912	5.0228	-1.1904	0.0898	1.9539
Feb 25	-1.9381	1.1024	1.6374	1.5535	1.6175	0.3566	6.6070	-1.4293	1.2649	1.5216
Mar 25	-2.6490	1.1374	0.7393	-0.0105	1.6585	0.8940	3.8326	-0.8888	-0.7077	3.5041
Apr 25	-1.6545	2.1385	0.0649	0.7612	3.9935	-2.1047	4.4488	-2.6568	-1.9089	4.9754
May 25	-1.7832	1.2159	-0.1087	1.7324	3.2795	-1.8708	4.3644	-2.9340	-1.0314	3.3724
Jun 25	-2.7935	1.6254	-0.5310	1.0283	2.9150	-1.9181	4.9921	-3.4469	-0.8007	1.7351
Jul 25	-1.3929	2.3276	1.2327	0.7181	3.7741	-1.8787	3.7752	-2.6091	0.5140	1.1178
Aug 25	-0.8800	1.4999	1.3337	0.0553	4.0791	-0.3407	4.7577	-1.9387	-0.9018	1.0339
Sep 25	-1.9590	2.9504	0.8588	0.8669	3.2699	2.0118	5.1792	-1.3210	-1.1122	1.4537
Oct 25	-0.1675	3.2789	1.1927	0.9835	3.6898	2.4708	4.8264	1.0711	-1.0535	0.2453
Nov 25	0.5783	2.0329	3.7083	0.9497	2.3625	0.8039	5.5015	1.0914	-0.7716	-1.7881
Dec 25	0.0859	1.8491	4.9959	2.8213	0.5717	0.4228	4.6335	1.8759	-0.4721	-1.8174
Jan 26	0.3469	2.8360	2.6156	3.0187	0.0952	2.4404	4.1969	0.3683	-0.8946	-0.5250
Feb 26	-0.5003	1.7813	1.5800	3.2957	-0.2889	1.9081	4.6893	0.9725	0.5239	2.1873
Mar 26	-0.3771	-0.3470	0.8078	1.5854	-0.5335	3.0729	3.3585	1.3843	0.7005	2.5630
Apr 26	-1.7156	0.3046	0.2418	1.5799	0.2557	1.2294	4.1990	0.2867	0.5942	3.5995
May 26	-4.2053	0.3618	0.0934	2.4833	1.9612	0.9619	4.6761	-0.9916	0.8650	1.6508
Jun 26	-4.7052	2.0525	0.8827	2.6592	1.5939	0.9129	2.6024	-0.9930	-1.0286	1.3358
Jul 26	-4.7537	2.0545	-0.1481	2.9090	-0.9654	2.2247	2.9258	-1.6532	-1.0798	0.3269
Aug 26	-2.1730	1.3915	-0.3431	0.5287	-1.3782	2.3301	1.8399	-2.1022	-0.2708	1.8946
Sep 26	-0.0317	1.8365	1.1863	2.2630	-0.3118	1.1710	1.0246	-0.8918	-2.1203	1.7760
Oct 26	1.2676	0.7621	1.5678	2.0988	-0.0632	2.4025	0.4582	0.3974	0.9389	1.5246
Nov 26	0.4786	1.8776	1.9978	1.8080	0.5812	2.1031	-0.7107	1.7116	-0.3342	2.6204
Dec 26	-0.1471	2.3312	1.5562	1.6493	2.3345	2.7583	-2.0724	2.8141	-0.7934	2.9072
Jan 27	-1.3148	4.3365	0.0019	3.7251	1.6716	2.9571	-0.3726	3.4119	-0.1542	1.7882

S4 Fig (panel a) illustrates a comparison between the forecasted percent weight gain values for four years and the original longitudinal data series using an ARIMAX model. The forecasted values exhibit variations when compared to the original data series. Notably, the forecasts suggest a distinct up-and-down pattern, indicating differences from the original data series that are likely attributed to exogenous factors such as water temperature and solar intensity (S4 Fig, panel b). These external variables are presumed to influence the observed fluctuations in percent weight gain, given that the pattern of the original data exhibits fluctuating or seasonal trends (S4 Fig, panel b). The forecasted values over three years exhibit a downward trend compare with the first year’s original data and, followed by an upward trend compare with the second- and third-years original data. It is concerning that seasonal change may be occurred and the percentage of weight gain may increase during the winter season. This assumes favorable climatic and water quality conditions, as well as other potential factors in the broodfish pond that could positively affect weight gain. This observed pattern raises concerns about the seasonal changes. While it may bring a positive impact on the sustainable aquaculture system in the long run, it also suggests the need for further investigation into the contributing factors.

## 4. Discussions

Tilapia, particularly the Nile tilapia (*O. niloticus*), is a globally cultivated fish species known for its rapid growth and adaptability, making it a favored choice in aquaculture operations across over 80 countries [75]. It thrives in high-density stocking and polyculture systems, including rice fields and shallow water bodies, where it provides ecological benefits by contributing nutrients to growing rice plants [75,76]. Robust broodfish are essential in fish hatcheries as they pass on superior genetic traits to their offspring, resulting in healthier, disease-resistant fish with higher reproductive success [77]. Enhancing the welfare of broodfish in pond settings leads to improved reproductive results and greater sustainability, thereby boosting the overall success of aquaculture operations [20,78]. Prioritizing the growth and health of tilapia broodfish is fundamental for sustaining high-quality seedlings and preserving genetic diversity, ultimately supporting responsible and sustainable broodstock management while boosting their commercial value in earthen ponds as a source of protein for local and global markets [7,21].

Contextually, our investigation enquires into the intricate relationships between climatic and water quality parameters and their profound implications for the growth of Tilapia (*O. niloticus*) broodfish. The significance of this study lies in its focused examination of how climatic variables—such as air temperature, humidity, rainfall, and solar intensity—and water quality parameters—like water temperature, dissolved oxygen, pH values, ammonia concentrations, and water transparency—collectively shape the growth patterns of Tilapia. Our exploration incorporates advanced modeling techniques, specifically ARIMA and ARIMAX, to depict the future real scenario of Tilapia broodfish production, with the aim of providing strategic insights for future planning. As we navigate through the observed fluctuations in these critical parameters, our goal is not only to enhance our understanding of Tilapia ecology but also to contribute valuable insights to the broader discourse on sustainable aquaculture practices and effective environmental management amid the challenges posed by variable climate and water conditions.

Our current three years’ study revealed fluctuations in climatic factors including air temperature (18.36°C to 30.48°C), humidity (46.61% to 85.80%), rainfall (0 mm to 485 mm), and solar intensity (3.02 to 7.9 hours). Similarly, water quality parameters exhibited variability, with water temperature (19.13°C to 33.20°C), dissolved oxygen (6.30 mg/L to 11.25 mg/L), pH values (6.74 to 10.32), ammonia concentrations (0 mg/L to 0.333 mg/L), and water transparency (15.37 cm to 27.32 cm) showing distinct fluctuations. Our study aligns with prior research, highlighting the intricate significant relationship between water temperature and Tilapia broodfish growth. Previous studies have emphasized the essential role of warmer air and discussed the potential impact it may have on dissolved oxygen levels, which are crucial for fish health [79–81]. There is a study that focuses on the importance of timing aquaculture operations with water temperature [82]. Additionally, the relationship between temperature and oxygen consumption, vital for aquaculture management, has been examined in several previous studies [83,84]. Rainfall, which can impact pH levels and consequently affect Tilapia well-being, has been discussed in a previous study [85,86]. The influence of solar intensity on ammonia concentrations has been explored in one study [87]. The effects of water de-stratification on the concentrations of dissolved oxygen and ammonia were examined in a previous study [88]. The study also highlighted the importance of water transparency for Tilapia, which is influenced by factors such as humidity and solar intensity [89,90]. Furthermore, the influence of light on fish behavior was discussed, emphasizing the significance of water transparency in aquaculture management [91].

Climatic factors wield a profound influence on fish growth within a pond, directly impacting crucial water quality parameters [76]. Fluctuations in air temperature, for example, lead to variations in water temperature—a critical factor influencing fish metabolic rates and behavior [92]. Rainfall patterns can alter pond pH, affecting the health of fish species [93]. Solar intensity plays a dual role, influencing both photosynthesis in phytoplankton and potentially triggering harmful algal blooms, impacting ammonia concentrations and overall water quality [94]. The interconnected dynamics between climatic variables and water quality parameters underscore the complexity of the aquatic environment and emphasize the need for comprehensive management strategies in aquaculture settings [95]. In our current study, we untied complex relationships through Pearson’s correlation analysis among percent length gain, percent weight gain, Feed Conversion Ratio (FCR), Specific Growth Rate (SGR), and water quality parameters. Percent length gain displayed refined very low negative correlations with water temperature (-0.063) and low correlation with pH (-0.378), while demonstrating a high positive correlation with ammonia (0.625) and moderate positive correlation water transparency (0.410). Similarly, percent weight gain showed very low negative correlations with water temperature (-0.044) and moderate negative correlation with pH (-0.523) and very low negative correlation with rainfall (-0.055) while higher positive correlations with ammonia (0.601) and percent length gain (0.765). FCR exhibited very low negative correlation with water temperature (-0.061), low correlation with solar intensity (-0.376) and pH (-0.338) while showed very low positive correlation with rainfall (0.245), DO (0.228) and percent weight gain (0.276). SGR exhibited moderate negative correlation with pH (-0.501) and moderate positive correlation with ammonia (0.450), higher positive correlation with percent length gain (0.635) and very high positive correlation with percent weight gain (0.968). It is to be mentioned that fluctuations in specific growth rate, percent length gain, percent weight gain, and FCR were strongly associated with the changes in water quality parameters, as strongly evidenced by Pearson’s correlation analysis conducted over three years course of the study. Different months exhibited variation of growth rates in terms of length and weight, with February standing out due to a substantial increase in length (3.931%) in February and percent weight gain (5.939%) in April, indicating favorable growth conditions during those periods. Conversely, November and December recorded the lowest percentages of length gain (0.212%) and weight gain (0.02%), respectively, implying less favorable conditions or management practices during those months. SGR, an essential indicator of growth efficiency, reached its highest value in April (0.193), suggesting a period of rapid growth possibly linked to favorable environmental factors and effective feed management. Conversely, May and December exhibited the lowest level of SGR (0.001) signals a notable growth decline, warranting further investigation. Moreover, the highest FCR (2.96) obtained in August, potentially indicating suboptimal feeding practices, while the lowest FCR (1.56) obtained in May signifies more efficient feed utilization. While fluctuations in certain growth parameters were influenced by changes in water quality parameters, the overall growth of tilapia exhibited a consistent upward exponential trend. Analyzing the factors influencing these fluctuations is essential for optimizing growth conditions and feed management in broodfish production. Our findings align with the study that suggests studying indeterminate-growing teleost fish for skeletal muscle aging research may offer unique therapeutic insights compared to current mammalian models [96]. Evidence from the Danioninae subfamily suggests that these fish possess adult muscle stem cells with greater proliferative capacity, possibly due to the involvement of Pax3/7 transcription factors, which might help some fish species delay or counteract muscle aging. Water quality parameters play a pivotal role in shaping fish growth and performance in aquaculture systems, with temperature influencing metabolism, oxygen levels supporting metabolic efficiency, and overall water quality affecting fish health and growth [97]. Additionally, the availability and quality of food sources, whether natural or provided feed, significantly impact SGR and FCR, with favorable conditions and proper feeding practices contributing to enhanced SGR and reduced FCR, thereby optimizing overall growth efficiency in fish farming operations [85]. We also observed a consistent exponential trend in the growth patterns of length and weight in tilapia. However, percent growth rate exhibited seasonal fluctuations attributed to the combined influence of climatic and water quality parameters, along with other factors presumed to be present in the pond ecosystem. The trends noted in total length, total weight, specific growth rate, percentage increase in length and weight, and FCR of tilapia broodfish are crucial topics for in-depth analysis and discussion. Our study consistently aligns with the findings of several previous studies explaining how the growth of Nile tilapia (*O. niloticus*) is influenced by various environmental factors, encompassing stocking densities, feed quality, culture systems, feeding practices, and several water quality parameters, including dissolved oxygen levels, salinity, water temperature, pH, as well as concentrations of ammonia, nitrite (NO_2_), and nitrate (NO_3_) [76,98]. In a comprehensive review article, it was stressed that a range of environmental factors significantly affects the growth performance of Nile tilapia [76]. Another study consistently identified an exponential growth trend in both length and weight for tilapia [99]. In a study, it was found that under a temperature of 22°C, all strains of Red, GIFT, and Supreme Nile tilapia exhibited similar exponential growth patterns, effectively conforming to the exponential growth model [98]. However, at 30°C, the GIFT and Supreme strains displayed exponential growth with significantly higher growth rates compared to the Red strain [76]. Temperature had a noteworthy impact on both the weight and age at the inflection point, influencing the descriptions of exponential growth for these tilapia strains and the quality of fits for the exponential growth model [98]. This variation in temperature also affected the dynamics of exponential growth in the batch. Our study supports previous studies that have emphasized the advantages of implementing effective management practices for conditioning tilapia broodstock and conducting mass spawning in hapas [100]. These practices have been shown to lead to enhanced growth rates in tilapia broodfish. Similarly, another research found that tilapia thrive in water with a pH range of 2.5 to 3.5 and a temperature between 74°F and 77°F [101]. Our study has provided valuable insights into the growth patterns of tilapia broodfish, notably the exponential growth observed in both total length and weight, indicative of favorable growth conditions. Fish growth in ponds depends on factors like nutrition, water temperature, efficient feeding, oxygen levels, water quality, stocking density, breeding, and health management [102]. Growth varies with genetics, environment, and species traits, fastest during early stages and slowing with maturity [76,102,103]. Fish growth can be categorized as either determinate, characterized by growth cessation at a certain life stage, or indeterminate, marked by continuous growth throughout their life [96,104]. Environmental factors like temperature, food, and water quality impact growth. Hyperplastic growth, characterized by cell division, is essential for the sustained growth of certain fish species such as tilapia. This type of growth is influenced by a combination of genetic factors, nutrient availability, and environmental conditions, enabling fish to adapt to varying circumstances [76,105,106]. A study was conducted to investigate the influence of earthen pond water physico-chemical parameters on the growth of Nile tilapia. The study was carried out in six earthen fish ponds using a semi-intensive culture system in Teso North Sub-County [85]. The study found that temperature and dissolved oxygen levels had a positive impact on the growth rate of tilapia, while conductivity, pH, and ammonia levels had a negative impact. The “Effect of Environmental Factors on Growth Performance of Nile Tilapia” is a review article that emphasizes the impact of various environmental factors on the growth performance criteria of Nile tilapia (*O. niloticus*) [76]. This article discusses the impact of managerial factors such as stocking density, food quality, culturing system, feeding frequency, and rate, as well as water quality parameters such as water dissolved oxygen, salinity, water temperature, pH, and ammonia, nitrite (NO_2_), and nitrate (NO_3_) concentrations. A previous study examined the effects of different environmental factors on the initial growth of Nile tilapia fry in a hapa-in-pond system [107]. The research findings indicated that water temperature, dissolved oxygen levels, and pH emerged as the most influential factors affecting the growth of Nile tilapia fry. This conclusion is supported by a study which investigated the length-weight relationship and condition factor of Nile tilapia (*O. niloticus*) in Lake Hawassa, Ethiopia [108]. Their study focused on assessing the length-weight relationship, population structure, and condition factor of Nile tilapia in the same location. Another study was conducted to compare the length-weight relationship and condition factor of *Tilapia zillii* and *O. urolepis* in both marine (Full Strength Sea Water-FSSW) and freshwater (FW) ponds [109]. Their main aim was to determine how the environmental conditions in these ponds influenced these two aspects for both species. Their results revealed that the “b” regression coefficient indicated negative allometric growth in FW and positive allometric growth in FSSW for both species. Specifically, *T. zillii* had an exponent “b” and condition factor (K) of 2.94 (3.3) and 2.07 (0.74) in FW and FSSW, respectively. In contrast, *O. urolepis* showed values of 2.81 (3.46) for “b” and 0.86 (0.53) for condition factor (K) in FW and FSSW, respectively.

ARIMA modeling and forecasting show significant potential for understanding and predicting fish growth dynamics, contingent upon factors such as data quality, stationarity, and the accurate selection of model orders [59]. When appropriately applied, ARIMA models at capturing linear patterns and trends within fish growth data, providing valuable insights for aquaculture and fisheries management [59,110]. In our study, we utilized three years longitudinal time series data to utilize ARIMA and ARIMAX models as an integrated approach to forecast the growth of tilapia in a traditional fish pond from February 2022 to January 2027. Our modeling and forecasting process involves several key steps, including model identification, parameter estimation, diagnostic testing of residuals, and forecasting. These steps are guided by previously established modeling procedures [110–113]. To assess data stationarity, we employed the ADF test, and in cases where the data exhibited non-stationarity, appropriate differentiation or transformation techniques were applied. Notably, the data in this particular series often did not contain any noise. This study recognized that an element’s influence on the accuracy of evaluations and the adequacy of findings was significant. The utilization of time series smoothing was viewed as a practical method for simplifying the process and reducing the influence of noisy elements. As a result, the data became more consistent and user-friendly. The selection ARIMA (3,0,3) for weight and ARIMAX (1,1,1) for percent weight gain as the optimal models was based on comprehensive statistical assessments and graphical analyses. These choices were substantiated by the observation of the lower normalized BIC and AIC values and the patterns observed in ACF and PACF plots, affirming the appropriateness of these models and their outcomes. Our findings are found consistent with previous studies, which have identified similar goodness-of-fit indicators in time series models, characterized by the lowest normalized BIC and AIC values, along with normally distributed residual ACF and PACF spikes [59,63,112,114–118]. Our analysis strengthens the appropriateness of ARIMA (3,0,3) for predicting weight and using ARIMAX (1,1,1) for predicting percent weight gain. This conclusion is based on a comprehensive evaluation of different metrics, including RMSE, MAPE, MaxAPE, MAE, MaxAE, Normalized Bayesian Information Criterion (BIC) and Akaike Information Criterion (AIC) values. Furthermore, the normal distribution of residual ACF and PACF spikes observed aligns with findings from numerous previous studies [110,119–122]. The forecasting trends of tilapia weight and percent weight gain obtained using the best fitted models ARIMA (3,0,3) and ARIMAX (1,1,1), respectively. The analysis of forecasting trend indicated a consistent upward pattern for weight and this denoted positive outcomes for future tilapia broodfish in the traditional pond system. On the other hand, forecasted values for percent weight gain exhibited seasonal critical up-down pattern and these behaviors represented significant decrease and increase pattern respectively, compared to the levels observed values in the past three years study. Similarly, the simulation results of our current study also revealed a distinct seasonal critical pattern in the percentage weight gain of tilapia broodfish over a three years period which is strongly support the findings obtained through ARIMAX (1,1,1) modeling and forecasting. Through seasonal variations, first year’s forecasting values exhibited lower pattern compare with the first-year original data series. Thereafter, the second- and third-years’ forecasted values exhibited seasonal upward pattern compare with the first- and second-years’ original data series which means that seasonal percent weight gain will be increased with the increase of tilapia broodfish weight. These are the eco-biological phenomena inside the traditional pond system where water temperature and solar intensity created strong influence as the potential exogenous factors on the variations of percent weight gain of tilapia broodfish.

Notably, there is a particular concern rises about the seasonal changes and forecast’s indication of a potential increase in percent weight gain during the winter season. This suggests seasonal changes and early broodfish development, which would have a positive impact on sustainable aquaculture practices. This emphasizes the need for further investigation into the implications of winter conditions on aquaculture systems and the identification of additional contributing factors of the broodfish pond. Despite the overarching exponential growth observed in tilapia, it is evident that the growth could be further enhanced if there were a consistent increase in percent weight throughout the year and seasonal influences from external factors were minimized. The positive route in tilapia growth indicates a promising prospect for broodfish production, with anticipated contributions to overall health and subsequent increases in fish production. Considering the densely populated landscape of Bangladesh and the continuous decrease in available land space caused by population growth, it is crucial to strategically increase tilapia production to meet the growing demand for nutritious fish. As such, our findings hold significant relevance for researchers, policymakers, and stakeholders seeking to develop effective strategies for tilapia broodfish, high-quality seed production, and the promotion of sustainable aquaculture to meet the future needs of a growing population.

We acknowledge the limitations in modeling and predicting the growth of tilapia broodfish by using ARIMA and ARIMAX with three years’ longitudinal time series data. Our approach focused on statistical modeling and its implications on biology to predict future scenarios. Our consideration of external influential factors, specifically water temperature and solar intensity on the pond water quality, is noted. It is crucial to recognize that our analysis did not encompass all other potential influencing factors in the pond system that could significantly impact growth and long-term trends. This is particularly important given the exponential nature of growth and its susceptibility to various external elements. Obtaining extensive time series data for robust modeling and forecasting posed challenges, especially in the pond for a long-term biological experiment conducted over an extended period in a specific location.

## 5. Conclusion

Our study assessed the influence of climatic and water quality parameters on the growth of tilapia broodfish, particularly their weight and percent weight gain, over a three-year period. The findings revealed a consistent exponential growth pattern in weight, significantly influenced by external factors such as water temperature and solar intensity. Using the ARIMA (3,0,3) model for weight and the ARIMAX (1,1,1) model for percent weight gain, we successfully forecasted tilapia growth from February 2024 to January 2027, predicting a 17.05% weight increase compared to January 2024. These models, despite certain limitations, have proven to be reliable forecasting tools. The predicted growth holds potential for advancing broodfish development, high-quality seed production, and promoting sustainable aquaculture practices in Bangladesh. These insights are crucial for decision-makers and stakeholders in formulating strategies to enhance sustainable fish production and address socio-economic and nutritional needs.

## Supporting information

S1 Fig Graphical expression of tilapia growth data. (Panels a & b) histogram of length & weight, for weight data exhibits normal distribution as a the curve is bell-shaped curve with symmetrical and has a distinctive peak in the center, gradually tapering off towards both ends, (panels b & c) individual value plot of length & weight, the density of dots on the plot reflects the frequency of fish at different length and weight values, with higher dot density indicating more common lengths and weights, while lower density points to less common measurements within the dataset. (panels c & d) box plot of length & weight. For length majority of the data points have lower values compared to the median as the median line in the box below the 50% mark which denotes a negative skew in the data distribution. But for weight majority of the data points have higher values compared to the median as the median line in the box upper the 50% mark which denotes a positive skew in the data distribution.
(TIF)

S2 Fig Pearson’s correlation. Matrix Plots of climatic variables, water quality and growth related parameters.
(TIF)

S3 Fig Cross correlation. (Panel a) % weight gain and water temperature and (panel b) % weight gain and solar intensity.
(TIF)

S4 Fig Comparison of forecasting and percent weight gain pattern with exogenous factors. Comparison of four-year forecasting values of percent weight gain with the original data series using ARIMAX (panel a), along with a comparative trend of water temperature, solar intensity and percent weight gain (panel b).
(TIF)
