# Supplementary figures and images for: Impact of climatic and water quality parameters on Tilapia (Oreochromis niloticus) broodfish growth: Integrating ARIMA and ARIMAX for precise modeling and forecasting
Source: PLoS One. 2025 Mar 13;20(3):e0313846. doi: 10.1371/journal.pone.0313846 (PMC11906076; doi:10.1371/journal.pone.0313846)

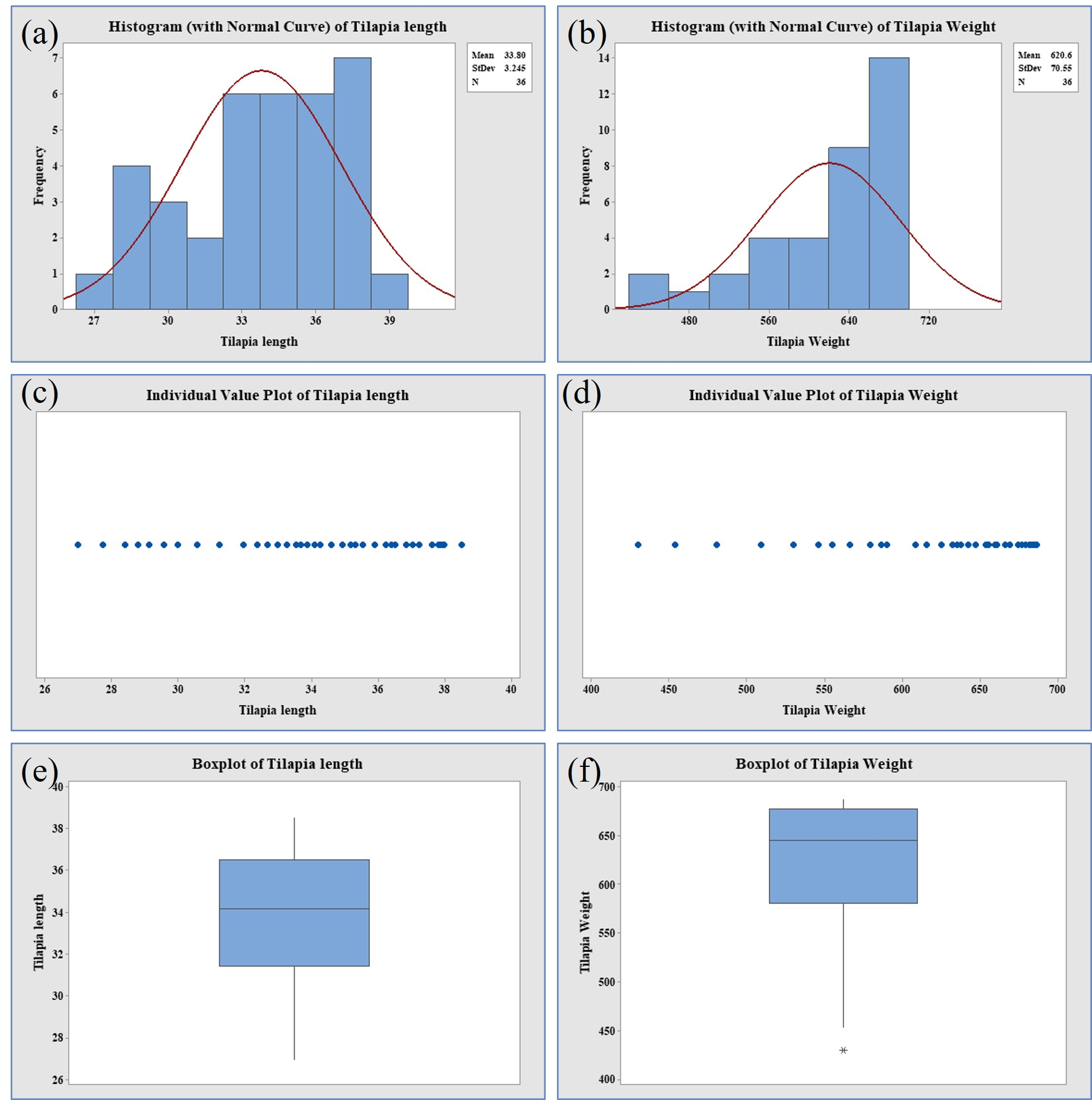

Supplement: S1 Fig — (Panels a & b) histogram of length & weight, for weight data exhibits normal distribution as a the curve is bell-shaped curve with symmetrical and has a distinctive peak in the center, gradually tapering off towards both ends, (panels b & c) individual value plot of length & weight, the density of dots on the plot reflects the frequency of fish at different length and weight values, with higher dot density indicating more common lengths and weights, while lower density points to less common measurements within the dataset. (panels c & d) box plot of length & weight. For length majority of the data points have lower values compared to the median as the median line in the box below the 50% mark which denotes a negative skew in the data distribution. But for weight majority of the data points have higher values compared to the median as the median line in the box upper the 50% mark which denotes a positive skew in the data distribution. (TIF) [file pone.0313846.s001.tif]

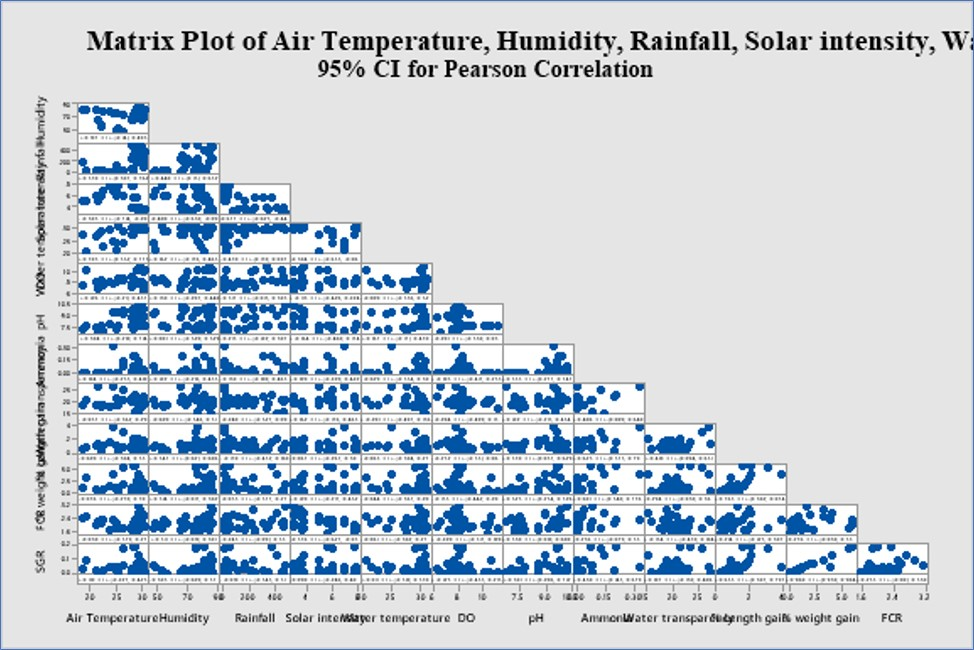

Supplement: S2 Fig — Matrix Plots of climatic variables, water quality and growth related parameters. (TIF) [file pone.0313846.s002.tif]

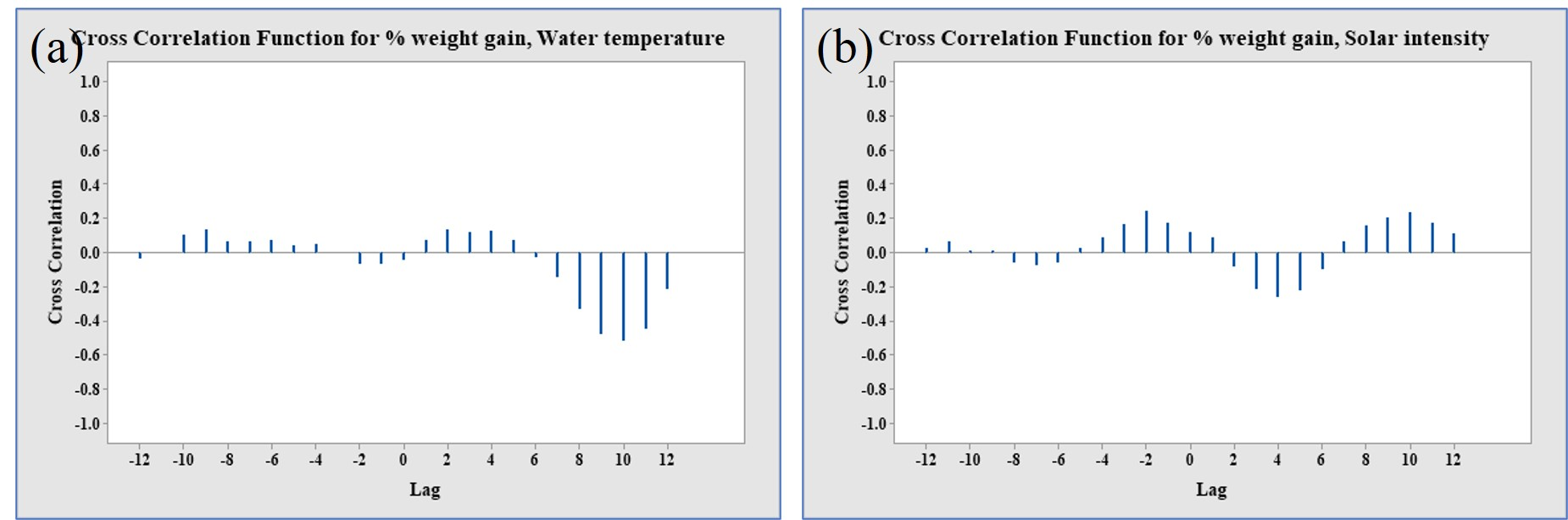

Supplement: S3 Fig — (Panel a) % weight gain and water temperature and (panel b) % weight gain and solar intensity. (TIF) [file pone.0313846.s003.tif]

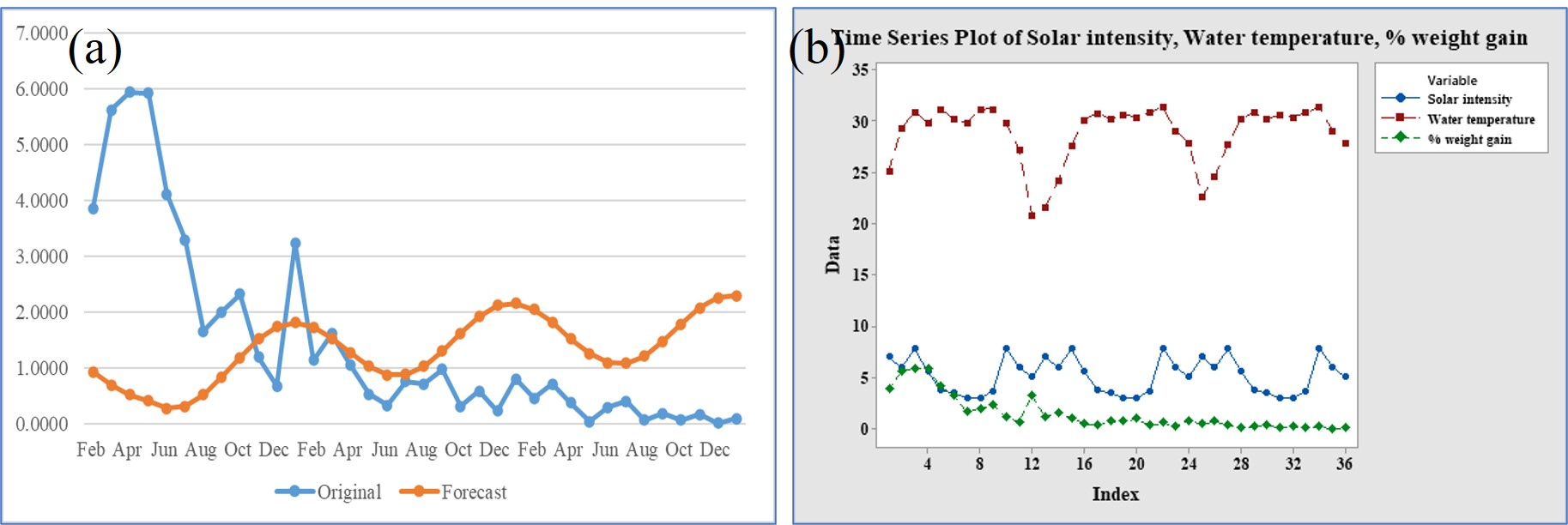

Supplement: S4 Fig — Comparison of four-year forecasting values of percent weight gain with the original data series using ARIMAX (panel a), along with a comparative trend of water temperature, solar intensity and percent weight gain (panel b). (TIF) [file pone.0313846.s004.tif]
